# Reduced Loss and Prevention of Substrate Modes with a Novel Coplanar Waveguide Based on Gap Waveguide Technology

**DOI:** 10.3390/s23062909

**Published:** 2023-03-07

**Authors:** Carlos Biurrun-Quel, Jorge Teniente, Carlos del-Río

**Affiliations:** 1 Antenna Group, Department of Electrical, Electronic and Communications, Public University of Navarra, 31006 Pamplona, Spain; 2 Institute of Smart Cities, Public University of Navarra, 31006 Pamplona, Spain

**Keywords:** gap waveguide, coplanar waveguide, transmission line theory, mmWave, electromagnetic bandgap, substrate modes

## Abstract

The Gap Waveguide technology utilizes an Artificial Magnetic Conductor (AMC) to prevent the propagation of electromagnetic (EM) waves under certain conditions, resulting in various gap waveguide configurations. In this study, a novel combination of Gap Waveguide technology and the traditional coplanar waveguide (CPW) transmission line is introduced, analyzed, and demonstrated experimentally for the first time. This new line is referred to as GapCPW. Closed-form expressions for its characteristic impedance and effective permittivity are derived using traditional conformal mapping techniques. Eigenmode simulations using finite-element analysis are then performed to assess its low dispersion and loss characteristics. The proposed line demonstrates an effective suppression of the substrate modes in fractional bandwidths up to 90%. In addition, simulations show that a reduction of up to 20% of the dielectric loss can be achieved with respect to the traditional CPW. These features depend on the dimensions of the line. The paper concludes with the fabrication of a prototype and validation of the simulation results in the W band (75–110 GHz).

## 1. Introduction

Coplanar waveguide (CPW) [1] is the core transmission line technology in the development of Monolithic Microwave Integrated Circuits (MMICS) as it facilitates the integration of active components by allocating its ground on the same face of the substrate as the signal traces. However, its operation at microwave, mmWave frequencies, and above is challenged by the propagation of substrate modes. In order to alleviate the detrimental effects of these modes, thinner substrates can be used [2], so that the higher order modes can be prevented from propagating. However, the TM_0_ mode is always present, as it has no cut-off frequency. Moreover, such thin substrates typically require some mechanical support which, if metallic, interacts with the line and modifies its arrangement of the EM fields, conforming a different transmission line known as Grounded CPW or Conductor-backed CPW (CB-CPW).

The presence of this lower ground plane presents some additional drawbacks, since parallel plate modes can be excited between this plane and the coplanar grounds on the opposite side of the substrate. This issue is typically prevented by periodically inserting some metallic vias along the perimeter of the line, balancing the electrical potential of both ground planes and preventing the leakage of power in the form of substrate modes. However, realizing these vias is not always a feasible option, either because the chosen substrate material is brittle and cannot be drilled, or because the vias drilling and metallization processes become highly complex and expensive.

In a recent work [3], we conceptualized a new transmission line technology to overcome these limitations—the Gap Coplanar Waveguide (GapCPW)—which combines the features of the Gap Waveguide (GW) technology with the conventional CPW. It consisted of a regular CPW on an electrically thin (≤$\lambda_{g}/4$) substrate being supported on top of a periodic structure that works as an Artificial Magnetic Conductor (AMC). Due to the definition of a stop band between the lateral grounds of the CPW and the AMC, the proposed GapCPW would be able to prevent the propagation of substrate modes in a relatively wide bandwidth (the bandwidth in which the periodic structure behaves as a PMC).

However, the concept of GapCPW has not been developed any further, and no experimental evidence of its operation has been provided yet. In this paper, our goal is to shed light onto this novel transmission line concept, by providing closed-form expressions for its electrical properties, studying the propagation in the line by means of Eigenmode simulations, and performing the first experimental validation of the concept with a working prototype at W band (75–110 GHz). As it will be detailed further in the following sections, the proposed GapCPW does not only prevent the propagation of substrate modes within a fractional bandwidth of up to 90%, but also is demonstrated to reduce the dielectric loss up to a 20% with respect to the CB-CPW under certain conditions.

## 2. Gap Waveguide Technology

**Figure 1:**
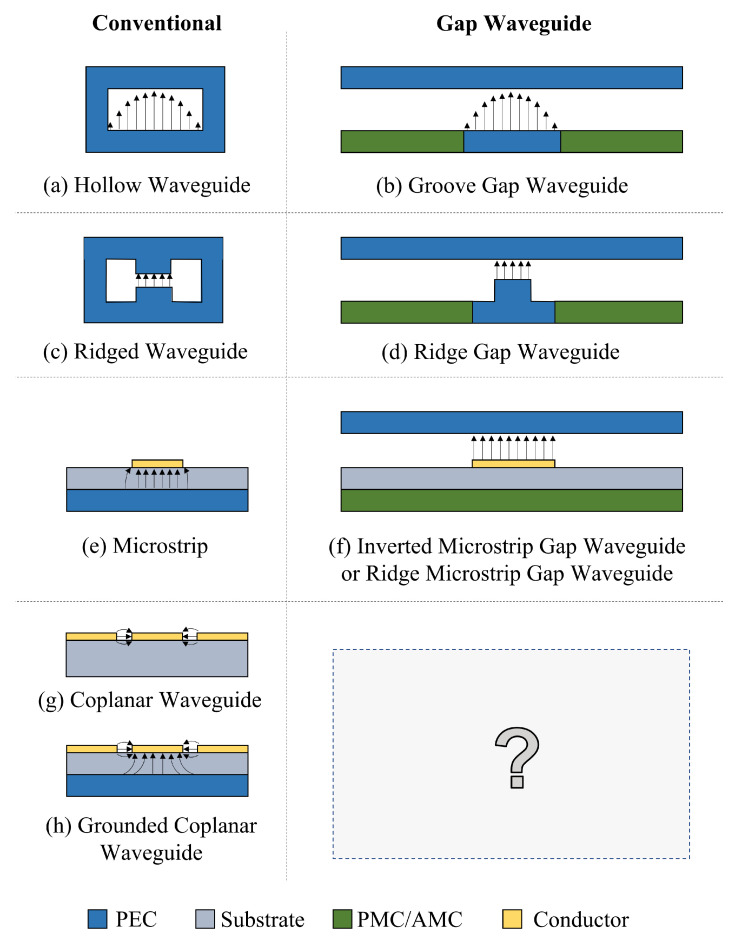
Summary of the typical transmission lines and waveguides together with their “Gap Waveguide” counterparts. E-field lines of the dominant mode are also plotted for each case. Blank spaces are considered air/vacuum.

**Figure 2:**
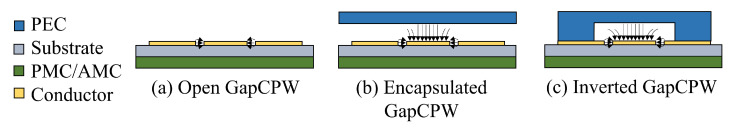
Proposed versions of the Gap Coplanar Waveguide. (**a**) GapCPW. (**b**) Encapsulated GapCPW. (**c**) Inverted GapCPW.

The Gap Waveguide technology relies on the fact that the propagation of electromagnetic waves between a Perfect Electric Conductor (PEC) plate and a Perfect Magnetic Conductor (PMC) plate is not possible if those plates are separated at a distance lower than a quarter of the wavelength [4]. Based on this feature, a plethora of GW technologies have been proposed in the last decades [5,6,7,8], all of which resemble a traditional waveguide/transmission line technology. This correspondence is shown in Figure 1. The realization of the PMC condition is typically achieved with artificial periodic structures that behave as a PMC, which are known as Artificial Magnetic Conductors (AMC), such as the bed of pins [9]. Looking at this figure, it is interesting to note that no CPW counterpart had been proposed in GW technology. The proposed GapCPW is illustrated in Figure 2 in three different configurations. The first configuration, (a), consists of an open GapCPW, which is essentially a CPW on a thin substrate supported by an AMC. If the substrate is thinner than a quarter of the guided wavelength in the dielectric slab, namely:

(1) $$h_{s}\leq \frac{\lambda }{4\sqrt{\epsilon_{r}}},$$

the region between the CPW conductors and the AMC creates a stop band for EM fields, preventing substrate modes from propagating and concentrating the E-field lines at the slots between the coplanar conductors. However, such an open line is vulnerable to external interference, and requires some sort of packaging/shielding. This shielding is represented in Figure 2b by a top PEC plate. This plate, if placed close enough to the central conductor of the CPW, may interact with it, creating a strong component over the air that resembles that of the inverted microstrip configuration [8]. Such propagation could be beneficial to overcome the losses due to the dielectric material. However, it is well known that the encapsulation of the planar technologies must be addressed properly. Otherwise, cavity modes may be excited, generating resonances and deteriorating the performance of the device. For instance, in Figure 2b, parallel plate modes could be excited between the top PEC plate and the CPW conductors. In order to overcome this limitation, packaging approaches relying on the use of AMCs [10,11] could be implemented.However, if a more compact solution is desired, the configuration in Figure 2c could be used. This configuration receives the name of Inverted Gap Coplanar Waveguide (IGCPW) and it includes a metallic encapsulation that is supported on top of the CPW, so that both lateral grounds are electrically connected by this encapsulation. The cavity generated inside is small enough to push the cut-off frequency of its cavity modes far beyond the frequency of operation. In addition, the electrical connection of both lateral grounds balances their electric potential, preventing the propagation of the typically undesired odd mode (also known as slotline mode). Therefore, this configuration pools the benefits of the previous configurations, at the same time it prevents the excitation of every unwanted mode in the line. Conversely, its realization might be more complex to achieve, as the dimensions of the requested channel are in the order of the dimensions of the CPW line.

The remainder of the paper is structured as follows: Section 3 provides closed-form expressions to extract the characteristic impedance and effective permittivity of the line in its different configurations. The propagation characteristics (i.e., Eigenmodes) of the line are then studied in Section 4 for the lossless scenario. Section 5 provides an in-depth insight into the loss taking place in the line, in comparison to the CB-CPW counterpart. The fabrication and experimental demonstration of a prototype operating in the W band (75–110 GHz) is addressed in Section 6. Further discussion of the results of the experiment are provided in Section 7, and conclusions and future research lines are highlighted in Section 8.

## 3. Closed-Form Expressions for Electrical Properties

**Figure 3:**
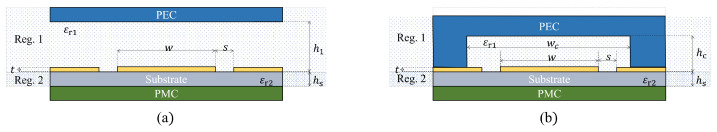
Geometry of the proposed lines. (**a**) Gap Coplanar Waveguide (GapCPW) with a possible top encapsulation. (**b**) Inverted Gap Coplanar Waveguide (IGCPW). Conductor thickness, *t*, is neglected in the analysis.

Conformal mapping has been employed in the past to obtain closed-form expressions for the relative effective permittivity ($\epsilon_{eff}$) and characteristic impedance ($Z_{0}$) of different CPW configurations [12,13,14,15,16]. This technique introduces a series of geometrical transformations to the cross-section of the structure to be analyzed, so that the resulting geometry is that of a parallel plates line, where the distance between the plates defines the capacitance per unit length. From this capacitance and the well-known expressions (where *L* is the inductance per unit length):

(2) $$v_{p}=\frac{1}{\sqrt{LC}}=\frac{c_{0}}{\sqrt{\epsilon_{eff}}}$$

and

(3) $$Z_{0}=\sqrt{\frac{L}{C}},$$

the following relationships can be obtained for $Z_{0}$ and $\epsilon_{eff}$ in terms of the capacitance per unit length of the line (*C*):

(4) $$Z_{0}=\frac{1}{c_{0}\sqrt{C\cdot C^{a}}},$$

(5) $$\epsilon_{eff}=\frac{C}{C^{a}},$$

where $C^{a}$ is the capacitance per unit length when all the dielectric materials in the geometry under study are replaced by vacuum. Since many transformations are already available in the literature, obtaining closed-form expressions of Equations (4) and (5) for the quasi-TEM even mode in the different GapCPW configurations in Figure 2 is a straightforward task. Such expressions are obtained relying on the assumption that the slots in the CPW are modeled as magnetic walls [15] (namely, that the E-field is parallel to the slots, and the H-field is perpendicular to it). In addition, the conductors are treated as infinitely thin PEC sheets. The geometries employed for this study are depicted in Figure 3, where both geometries shown are valid for the encapsulated version (Figure 2b) if $w_{c}\to \infty$ and $h_{1}$ = $h_{c}$. Each line is analyzed by distinguishing two regions, each one with a different permittivity. Namely, Region 1 includes the vacuum-filled part over the substrate, with $\epsilon_{r}$ = 1, whereas Region 2 concerns the substrate in between the CPW metallization and the PMC ground plane, characterized by $\epsilon_{r,i}$. The partial capacitance of each region is computed as

(6) $$C_{i}=2\epsilon_{0}\epsilon_{r,i}\cdot \frac{K(k_{i})}{K(k_{i}^{\prime })},$$

where $K(k_{i})$ and $K(k_{i}^{\prime })$ are the complete elliptic integrals of the first kind with modulus $k_{i}$ and $k_{i}^{\prime }$ is the complement, defined as $k_{i}^{\prime }=\sqrt{1-k_{i}^{2}}$. These modules result from the conformal mapping transformations, to which the reader is referred to [16] for a more detailed mathematical description. The partial capacitances of each region are calculated and added together to obtain the total capacitance of the line per unit length, which is then inserted in Equations (4) and (5).

### 3.1. Open/Encapsulated Gap Coplanar Waveguide

To analyze this geometry, the expressions developed in [15] for the even mode can be reused. There, the authors analyzed a Broadside-Coupled CPW, which is a symmetric line on both sides of the substrate. As a result, the analysis of its even mode assumes a magnetic wall symmetric boundary, making it electrically equivalent to the line proposed here. As provided in [15], the expressions for these modules are:

(7) $$k_{1}=\frac{\tanh\left(\frac{\pi w}{4h_{1}}\right)}{\tanh\left(\frac{\pi (w+2s)}{4h_{1}}\right)},$$

(8) $$k_{2}=\frac{\sinh\left(\frac{\pi w}{4h_{1}}\right)}{\sinh\left(\frac{\pi (w+2s)}{4h_{1}}\right)}.$$

Here, the assumption of an open line ($h_{1}\to \infty$) reduces Equation (7) to:

(9) $$\lim_{h_{1}\to \infty }k_{1}=\frac{w}{w+2s}$$

### 3.2. Inverted Gap Coplanar Waveguide

As for the geometry in Figure 3b, it can be checked that Region 2 is similar to the previous geometry, thus $k_{2}$ can be computed with Equation (8) as well. Conversely, Region 1 includes a metalized channel of width $w_{c}$ and height $h_{c}$, which requires a different geometrical transformation. Such transformation was developed in [17] for “micro-shield lines”, and the modulus of the elliptic integral can be obtained following:

(10) $$k_{1}=\frac{sn\left(\frac{w}{2\beta }\right)}{sn\left(\frac{w+2s}{2\beta }\right)},$$

(11) $$\beta =\frac{w_{c}}{2\cdot K(\gamma )}\;\mathrm{and}\;\gamma ={\left[\frac{e^{\frac{\pi w_{c}}{2h_{c}}}-2}{e^{\frac{\pi w_{c}}{2h_{c}}}+2}\right]}^{2}$$

where sn(x) is the Jacobi elliptic sine, and β and the modulus γ have been kept as in the original notation and have no relationship with the phase or the propagation constant in the line, traditionally represented by these symbols. In addition, it can be checked that, if $w_{c}\to \infty$, Equation (10) is reduced to the previous case, (7), for the encapsulated GapCPW.

### 3.3. Validation

**Figure 4:**
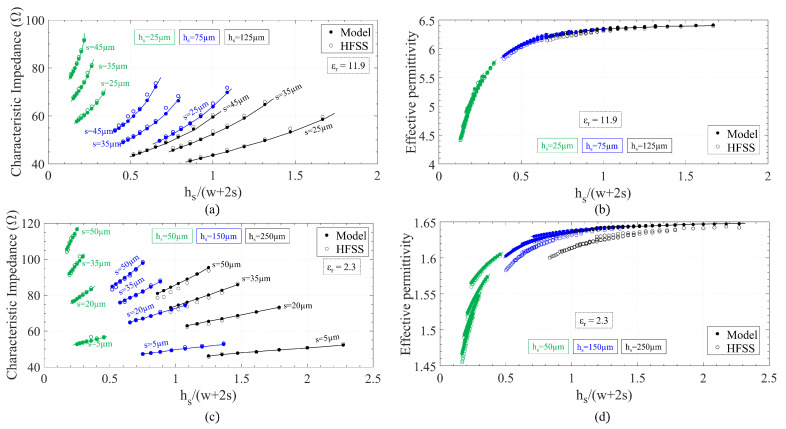
Comparison between closed-form expressions and finite element simulations for the open GapCPW line. (**a**) $Z_{0}$ for high-permittivity substrate. (**b**) $\epsilon_{eff}$ for high-permittivity substrate. (**c**) $Z_{0}$ for low-permittivity substrate. (**d**) $\epsilon_{eff}$ for low-permittivity substrate.

In order to validate these closed-form expressions, a series of simulations were performed with ANSYS Electromagnetics Desktop (HFSS) for a plethora of combinations of the line dimensions. Two different permittivities, 2.3 and 11.9, were considered. The simulations were performed by setting wave ports at both ends of a GapCPW section with a perfect PMC bottom ground. Figure 4 shows a comparison of the values obtained by HFSS (empty markers) and the ones calculated with the following expressions (filled markers) for the open GapCPW. Overall, an excellent agreement is found, with deviations of less than 3%.

**Figure 5:**
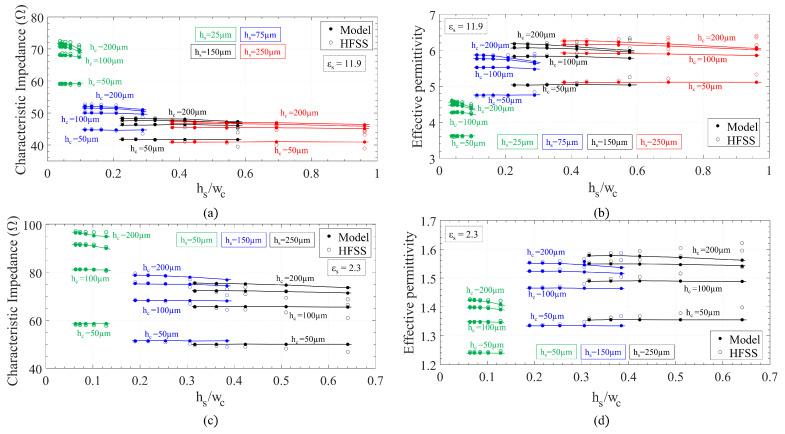
Comparison between closed-form expressions and finite element simulations for the IGCPW line. (**a**) $Z_{0}$ for high-permittivity substrate. (**b**) $\epsilon_{eff}$ for high-permittivity substrate. (**c**) $Z_{0}$ for low-permittivity substrate. (**d**) $\epsilon_{eff}$ for low-permittivity substrate.

The general trend is that a larger slot size increases the impedance of the line, decreasing its permittivity slightly. Conversely, a larger central conductor width follows the opposite trend. In addition, increasing the substrate height reduces the characteristic impedance while increasing the effective permittivity. A similar procedure was followed to validate the expressions for the IGCPW (Figure 5). In this case, only the dimensions of the metallic channel ($w_{c}$, $h_{c}$) and the substrate height ($h_{s}$) are swept, since the rest of the parameters had the same influence as in the open GapCPW. A central conductor width of 190 μm and slot size of 50 μm were chosen for the study on the low-permittivity substrate, whereas 80 μm and 40 μm were, respectively, selected for the high-permittivity substrate. A remarkable agreement is found in this case as well. However, the expressions provided seem to overestimate $Z_{0}$ and underestimate $\epsilon_{eff}$ for lower values of $w_{c}$, a trend which is remarkably significant for higher $h_{s}$. This finding is explained by the fact that a narrower channel interacts with the slots in the coplanar waveguide (the smallest $w_{c}$ considered was just 50 μm broader than the CPW, (*w* + 2*s*)). As a result of this interaction, the assumption of the slots behaving as magnetic walls is no longer applicable. In conclusion, it can be observed that a lower channel height, $h_{c}$, provides a higher characteristic impedance while reducing the effective permittivity. As for the channel width, its influence is almost negligible, provided that it is wide enough not to interact with the slots. Nevertheless, its width will play a key role in defining the cut-off frequency of the undesired odd mode, a matter which is addressed in the next section.

## 4. Modes and Propagation in GapCPW

This section aims to provide a deeper insight into the modes propagating in the different GapCPW configurations presented earlier. For this purpose, the eigenmode simulator available in HFSS has been employed to obtain the dispersion diagrams of each line and the influence of some of its dimensions. Nevertheless, before tackling this goal, the same eigenmode simulator is used to analyze the propagation in the conventional CB-CPW to identify the previously commented issues regarding the propagation of substrate modes. For these studies, a 100 μm-thick silicon substrate ($\epsilon_{r}$ = 11.9) will be considered, targeting a characteristic impedance of 50 Ω (which, employing the expressions from the previous section, can be obtained approximately with *w* = 80 μm, *s* = 40 μm).

### 4.1. Conductor-Backed CPW

**Figure 6:**
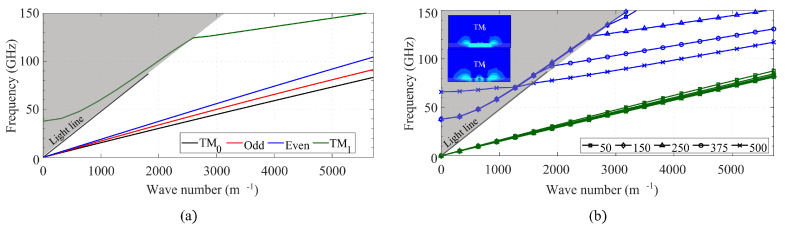
Dispersion curves in a CB-CPW on 100 μm silicon substrate. (**a**) First 4 modes where lateral ground extension is 250 μm. (**b**) Substrate modes (TM_0_—green, TM_1_—blue) for different lateral ground extensions (in μm). Insets: E-field magnitude of the corresponding modes.

Figure 6a shows the dispersion diagram of a CB-CPW with a lateral ground extension of 250 μm, computed with HFSS. Here, three different modes start propagating without any cut-off frequency: the unavoidable TM_0_ substrate mode, the odd mode (slotline mode), and the quasi-TEM even (desired) coplanar mode. In addition, at approximately 123 GHz the next substrate mode, TM_1_, starts propagating. However, the cut-off frequency of this mode is strongly dependent on the lateral ground extension. This is shown in Figure 6b, which provides the dispersion curves of both TM_0_ and TM_1_ modes for different lateral ground extensions. Here, it can be seen that the cut-off frequency of the TM_0_ remains unchanged, although the higher ground extension implies a higher effective permittivity of the mode (the slope in the curves is lower). On the other hand, the cut-off frequency of the TM_1_ does show a strong dependence on the lateral ground size. These findings are in agreement with [18], where substrate modes in different CPW topologies are also analyzed with similar conclusions.

### 4.2. Open Gap Coplanar Waveguide

**Figure 7:**
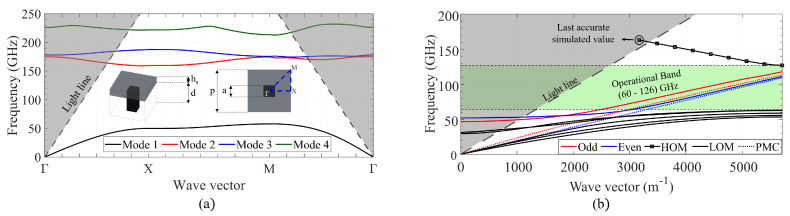
Dispersion diagrams. (**a**) Periodic bed of rectangular pins with a sketch of the unit cell dimensions. (**b**) Gap Coplanar Waveguide on 100 μm silicon for a 1 mm lateral grounds extension. HOM: High Order Mode. LOM: Low Order Modes. Green area: bandwidth of operation.

The first step to analyze the propagation in the proposed GapCPW is to design the AMC structure to be placed below the substrate. These AMCs are typically realized with periodic textured structures. Although a plethora of structures has been studied in recent decades [5,7,9,19,20,21,22,23,24], in this case, the traditional bed of metallic pins was selected because it is the most widely used structure employed in Gap Waveguide technology. The chosen dimensions were a period *p* of 550 μm, a pin width *a* of 175 μm, and pin height *d* of 350 μm. Its dispersion diagram is shown in Figure 7a, together with a sketch of the unit cell (single pin). The targeted frequency band was the W band (75–110 GHz). A band gap of roughly 100 GHz (from 58 to 158) is observed between the first two modes, comprehending the whole targeted band. The band gap between 188 GHz and 212 GHz will not be taken into consideration to focus on the previously selected frequency band.

**Figure 8:**
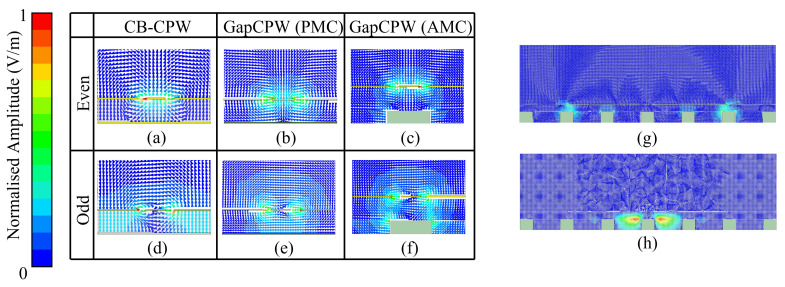
Screenshots from HFSS Eigenmode simulator concerning the E-field arrangement in different modes. (**a**–**c**) coplanar even mode for different bottom boundary conditions: PEC (CB-CPW), PMC, and AMC, respectively. (**d**–**f**) coplanar odd (slotline) mode. (**g**) E-field arrangement for the highest LOM (low order mode) at the GapCPW. (**h**) E-field arrangement for the lowest HOM (high order mode) in the GapCPW.

Once designed, the AMC was integrated below a CPW with the same dimensions ($w,s,h_{s}$) as the previous CB-CPW. The resulting dispersion diagram is provided in Figure 7b. Here, a wide band between 60 and roughly 126 GHz is observable, where only the odd (red) and even (blue) coplanar modes are propagated. In addition, the curves corresponding to these modes when the AMC is replaced by a PMC sheet in the simulator are also included (dotted lines). The curves corresponding to the even mode agree remarkably well, showing that the AMC does behave like a PMC within its bandwidth of operation. On the other hand, the discrepancy between the curves concerning the odd mode is attributed to a higher interaction with the pin structure. This can be observed in Figure 8, where some screenshots from the eigenmode simulator are provided. The fact that the curve corresponding to the High Order Mode is not continued over the light line is because the data points calculated by the software were not stable enough (presumably because in the region above the light line the modes are being radiated and many modes were being handled by the simulator).

The diagram in Figure 7b was computed for a lateral ground extension of 1 mm on each side. The influence of this dimension was studied more in–depth, since the bed of pins requires a PEC plane on top to effectively create a stop band for the substrate modes. Table 1 includes a summary of such analysis. Overall, it is concluded that the size of the lateral grounds affects the bandwidth of operation, with special influence on the lower boundary of the band. Furthermore, it can be concluded that at least one period (550 μm) of ground on each side is required in order not to reduce the bandwidth of operation dramatically. However, it would be recommended to use at least two or three periods to maximize the bandwidth. Another observable fact is that increasing the extension of the ground increases the effective permittivity of the CB-CPW, whereas such an increase reduces the effective permittivity of the GapCPW. This is reasonable, as in the CB-CPW a larger ground plate will allow a higher portion of the fields to propagate inside the substrate, whereas in the GapCPW a larger ground plate will enhance the definition of the stop band.

### 4.3. Inverted Gap Coplanar Waveguide

**Figure 9:**
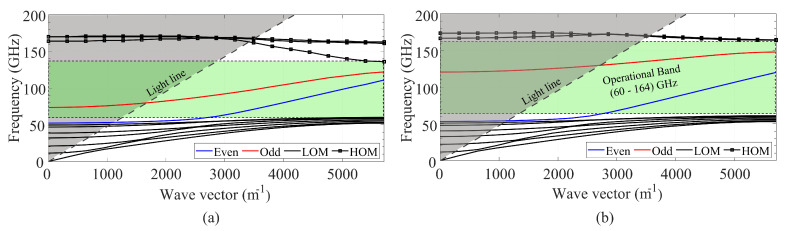
IGCPW dispersion diagrams for varying channel dimensions. (**a**) $w_{c}$ = 450 μm, $h_{c}$ = 150 μm. (**b**) $w_{c}$ = 350 μm, $h_{c}$ = 50 μm.

The same procedure was followed to study the IGCPW configuration. Figure 9a shows the dispersion diagram for a IGCPW line with a 450-μm-wide, 150-μm-high channel. It can be observed that the operational band is enlarged with respect to the previous configuration, especially concerning the upper boundary. The cut-off frequency of the odd mode is also pushed higher in frequency, up to roughly 80 GHz, whereas in the open GapCPW, this frequency was about 45 GHz. This gives evidence of the influence of the channel dimensions in the prevention of the odd mode (as well as in the operational band). To illustrate with another example, Figure 9b shows the dispersion diagram corresponding to a channel width of 350 μm and a height of 50 μm, where the cut-off frequency of the odd mode is pushed away from the W band), up to 130 GHz. In addition, the operational band (substrate-mode free band) is increased up to more than 160 GHz. Table 2 summarizes the influence of the channel dimensions on the propagation inside the line. Overall, it shows how both channel dimensions are involved in the determination of $f_{odd}$, though $h_{c}$ seems to play a major role. Whereas fixing $h_{c}$ and sweeping $w_{c}$ results in a variation of $f_{odd}$ between 19 and 28%, sweeping $h_{c}$ for a fixed width varies this frequency up to 90%. In addition, it can be checked that the lower bound of the band is not affected by the channel dimensions (the differences between the displayed values are due to the accuracy of the simulations), whereas the higher end of the band increases for a reduced channel size. Lastly, the simulations showed how a smaller channel reduces the effective permittivity of the odd mode in the line. This finding is in agreement with the closed-form expressions presented in the previous section, and it is due to a higher interaction of the metallic encapsulation with the central conductor, giving rise to a strong E-field component propagating over the air inside the channel.

## 5. Reduced Loss in GapCPW

The preceding sections have presented an in-depth eigenmode analysis of the newly proposed Gap Coplanar Waveguide and its “Inverted” counterpart. Both configurations have been demonstrated to effectively suppress substrate modes in the relevant frequency band. It was also noted that these lines have a lower relative permittivity than the conventional CB-CPW, indicating that a higher proportion of the electromagnetic fields are propagated through the air, potentially leading to lower dielectric losses. However, the presence of additional conductors, such as a bed of pins or metallic encapsulation, may also introduce additional loss mechanisms, especially at higher frequencies. The previous analysis was performed using perfect electric conductors (PEC) and lossless silicon, resulting in purely real eigenvalues and no attenuation. In this section, each individual loss source will be analyzed. The derivation of the attenuation constant (α) from the computed eigenmodes is achieved following the procedure presented in [25] for uniform structures.

### 5.1. Conductor Loss

The conductor loss was assessed individually for (i) the coplanar conductors, (ii) the backside metallization (CB-CPW) or bed of pins (GapCPW), and (iii) the metallic encapsulation (IGCPW). The coplanar conductors were designed as 2-μm-thick copper sheets with a conductivity of 58 MS/m, which is approximately 8 to 10 times higher than the skin depth of copper in the targeted frequency band. However, it is important to note that the actual conductivity in a practical scenario may be lower due to two main factors: a decrease in copper’s conductivity in the THz regime, as reported in [26], and a reduction in effective conductivity due to surface roughness, as described in [27]. The AMC, the metallic cover of the IGCPW, and the bottom ground of the CB-CPW were simulated as aluminum with electrical conductivity of 38 MS/m, as they would result from a typical CNC manufacturing process used in their manufacturing.

**Figure 10:**
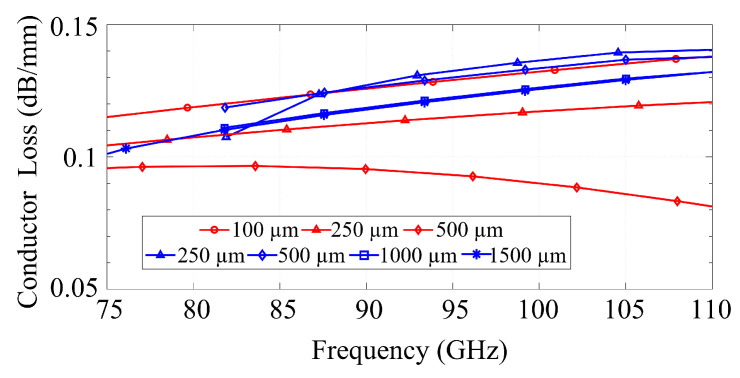
Comparison of conductor loss associated to a 2-μm-thick metallization of copper with finite conductivity (58 MS/m) in CB-CPW (red) and GapCPW (blue).

Figure 10 presents the losses of the coplanar metallization for both the CB-CPW (in red) and the GapCPW (in blue). It can be seen that the loss of the CB-CPW decreases with an increase in the lateral extension of the ground. This agrees with previous research on finite ground CPWs [13,28]. In the case of the GapCPW, the loss is slightly higher for smaller ground extensions but decreases as the ground size increases, saturating when at least two periods of AMC/PEC are covered (1000 μm on each side). Overall, the loss due to the coplanar conductors remains within the same order of magnitude for both lines.

**Figure 11:**
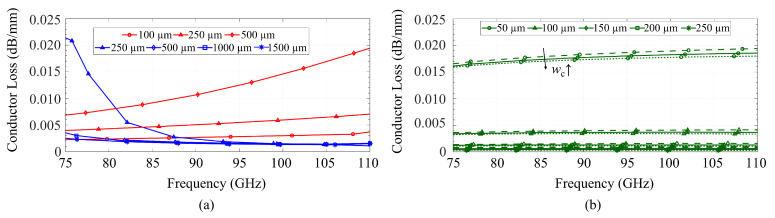
Conductor loss associated with (**a**) the back metallization and (**b**) top encapsulation. Aluminum is considered in every case. Red: CB-CPW. Blue: GapCPW. Green: IGCPW. Channel width in (**b**) is represented by dashed lines (350 μm), solid lines (450 μm) and dotted lines (550 μm).

Figure 11a compares the loss of the bottom ground of the CB-CPW and the AMC. Since the majority of the fields are concentrated around the slots in the CPW in the GapCPW, the loss of the AMC is nearly negligible and, similarly to Figure 10, this loss is independent of the lateral ground extension, as long as it is broad enough to define the PEC/PMC condition. It can also be seen that the loss increases at the lower frequency for the GapCPW with 250 μm grounds, due to a higher interaction with the AMC when the stopband is not defined. Conversely, the CB-CPW experiences an increase in loss with higher ground extensions, although this loss is significantly smaller compared to the conductor loss shown in Figure 10. Figure 11b compares the losses associated with the metallic cover of the IGCPW for different channel dimensions. It can be seen that lower channel heights result in a higher loss, which is due to the stronger interaction with the top encapsulation. On the other hand, this loss becomes negligible when the channel height exceeds 150 μm. In terms of channel width, a smaller $w_{c}$ (350 μm, dashed line) incurs in a relatively slightly higher loss, but its influence becomes insignificant for higher channel heights. This is, once again, due to the stronger interaction with the top encapsulation for smaller channel dimensions.

### 5.2. Dielectric Loss

The dielectric loss is directly related to the imaginary part of the electric permittivity of the dielectric material, which is dependent on its electrical conductivity:

(12) $$\hat{\epsilon }=\epsilon^{\prime }-i\frac{\sigma }{\omega }$$

**Figure 12:**
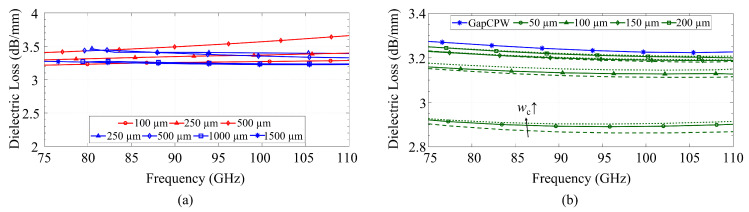
Dielectric loss associated with a silicon substrate with a resistivity of 10 Ω·cm. (**a**) Comparison between CB-CPW (red) and GapCPW(blue) for different lateral ground extension. (**b**) Comparison between GapCPW (blue) and IGCPW (green) for different channel heights and widths. Channel width in (**b**) is represented by dashed lines (350 μm), solid lines (450 μm) and dotted lines (550 μm).

In this paper, a silicon substrate was chosen to perform the analysis of losses. However, the study performed here would be applicable to any other substrate material with known conductivity (or, equivalently, with a known tangent of loss, tanδ). Commercially available silicon substrates may present different doping concentrations and as a result, one may find resistivities from below 1 Ω·cm up to 10 kΩ·cm, or even higher, experimenting lower losses for higher resistivities. The resistivity of the silicon substrate in the simulation was swept for both the CB-CPW and the GapCPW between the aforementioned values. However, it was found that, because it was the only loss mechanism taking place, the computed attenuation constant was directly proportional to the chosen resistivity. Figure 12a shows the loss for the case of 10 Ω·cm, for the even mode in CB-CPW (red) and open GapCPW (blue). The red and blue curves are provided for a varying lateral ground extension. Note that the blue curves are not continued below the cut-off frequency. By looking at these curves, it can be concluded that the larger the lateral ground in the CB-CPW, the larger the dielectric loss is. This is explained by a larger proportion of the fields being propagated inside the substrate for larger lateral grounds and higher frequencies. This reasoning also supports the reduced conductor loss observed in Figure 10 for the 500 μm red curve. Conversely, an increasing lateral ground extension in the GapCPW provides a reduction in dielectric loss because the stopband between the AMC and the grounds is more strongly defined (recall Table 1). For ground extensions larger than 2*p*, the dielectric loss reaches its minimum, being lower than in the CB-CPW counterpart. The reason why the loss is higher for the lower GND extensions is that the stopband between the AMC and the CPW conductors is not fully defined. Therefore, the EM fields in between the slots interact with the pins, thereby propagating also inside the substrate. All in all, it is seen that a reduction of up to 10% in the dielectric loss can be achieved with respect to the CB-CPW when the lateral grounds are wide. Otherwise, the dielectric loss lies within the same order of magnitude in both lines.

In addition, the IGCPW (green) was simulated for varying channel dimensions and compared with the loss in the GapCPW with 1500 μm lateral ground. This comparison is shown in Figure 12b. Here, it is shown how a lower channel height provides a lower substrate loss, a finding supported by the idea of a higher interaction between the coplanar conductors and the top encapsulation, in a similar way to Figure 11. This loss becomes increasingly higher for greater channel heights, until the top encapsulation is distant enough, providing an attenuation equivalent to that of the GapCPW. For the smaller channel dimensions, an additional 10% dielectric loss reduction can be achieved with respect to the open GapCPW. This reduction will be higher if compared with open GapCPW with smaller ground planes.

## 6. Experimental Validation

Up to this point, a comprehensive study of the open GapCPW and the IGCPW has been carried out by means of eigenmode simulations to provide evidence that the dielectric loss is reduced in these configurations and that the substrate modes are prevented within a wide bandwidth. The intermediate version in Figure 2 has been left out of this analysis, as it corresponds to an IGCPW with an infinitely wide channel and its behavior can be inferred from the previous study. In this section, we detail the procedure followed to experimentally validate the GapCPW concept, including the design of the AMC and the lines, their fabrication, and the measurement campaign.

### 6.1. Design

The targeted frequency band for our experiment was the same W band employed before. Likewise, the chosen substrate was a 100-μm-thick silicon wafer, whose resistivity, ρ, was unspecified between the range 0.1 and 30 Ω·cm, as stated by the supplier. Whereas this resistivity would not be practical in an actual scenario, the relatively high loss in dB/mm of this material allowed us to clearly identify an improvement in the measured losses without the need for fabricating excessively long lines. The same AMC structure considered in the previous analysis was also used (p = 550 μm, a = 175 μm, and d = 350 μm). This AMC was originally designed following the guidelines for designing an optimal bed of pins described in [7], which can be used for any other frequency band and material, as long as the condition in Equation (1) is satisfied. A set of lines with different lengths and a targeted impedance of 50 Ω was designed following the expressions in Section 3. In addition, an on-wafer custom TRL (Thru-Reflect-Line) [29] was designed and included on the same wafer, to be able to de-embed the effect of the Ground–Signal–Ground probes employed in the measurement.

### 6.2. Fabrication

**Figure 13:**
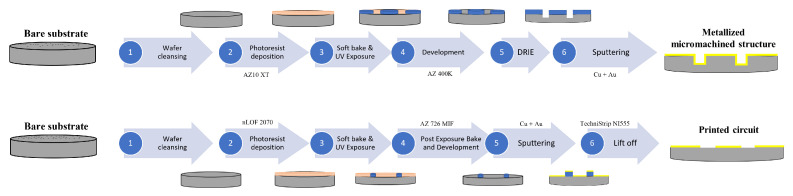
Diagram of the fabrication process followed in this work. **Top**: Fabrication of the AMC structure. **Bottom**: Fabrication of the coplanar lines.

**Figure 14:**
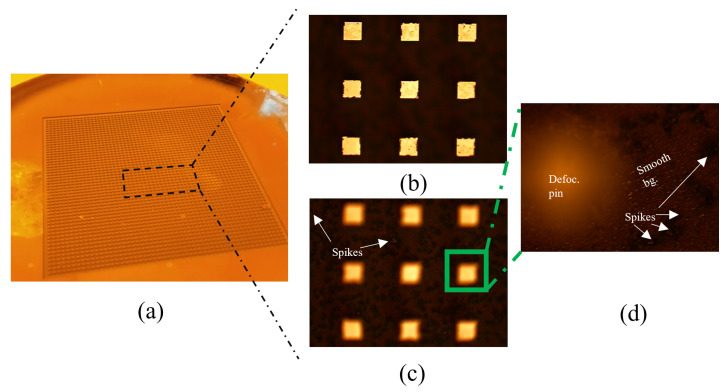
Bed of pins manufactured by DRIE Bosch process and coated with copper and gold by sputtering process. (**a**) Overview. (**b**) Front view with optical focus on pin layout. (**c**) Front view with optical focus on background (bg) surface. (**d**) Zoomed view of background surface.

**Figure 15:**
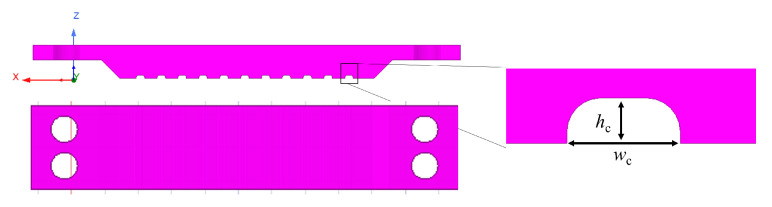
Geometrical sketch of the micromachined aluminum encapsulations to implement the IGCPW.

The CNC machining of the periodic bed of pins was challenging due to the small structure size and manufacturing tolerances, requiring commissioning to a third-party workshop. Instead, we manufactured it in-house using silicon micromachining with photolithography techniques and a Deep Reactive Ion Etching (DRIE) Bosch process. The fabrication process carried out in our clean room facilities is sketched in Figure 13. The resulting bed of pins, shown in Figure 14a, had some inhomogeneities, with some spikes of less than 50 μm being randomly generated during the DRIE process for undetermined reasons, but overall met the desired dimensions, with an excellent agreement in the pin section and a deviation in the pin height of about ±10 μm. The AMC was then metalized with 2 μm of copper and about 50 nm of gold (to prevent the oxidation of the former). A set of coplanar lines with different lengths and lateral ground extensions, as well as customized TRL kits, were designed for a single-run photolithography process. The metallization process was the same as in the AMC. In addition, some shielding structures were CNC-machined on Aluminum, containing carved channels of 450 μm × 150 μm (Figure 15), to test the IGCPW concept. In addition, an aluminum block was also CNC-machined to accommodate the bed of pins and substrate wafers, facilitating the measuring tasks.

### 6.3. Measurements

**Figure 16:**
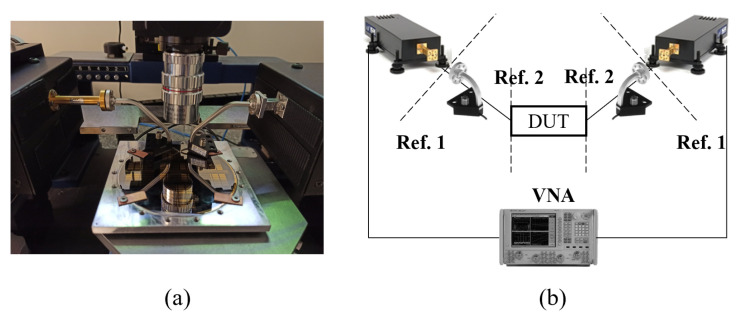
(**a**) Measurement set-up. (**b**) Two-tier de-embedding TRL procedure with reference planes.

The measurement setup, consisting of a Keysight PNA-X N5242A VNA, two Virginia Diodes WR-10-VNAX T/R waveguide extenders, two Picoprobe GSG-120 coplanar probes with rectangular waveguide output, and the Cascade Microtech EPS150MMW probe station, is shown in Figure 16. The calibration of the system was performed in two differentiated steps. First, a semi-automatic waveguide TRL calibration was performed with the VNA calibration wizard and the waveguide extenders with VDI’s available calibration kit, establishing the measurement reference plane to their waveguide output. By eliminating the influence of the waveguide extenders with the first-tier TRL, we could increase our confidence in the de-embedded results after the second TRL step. To perform this step, the different on-wafer customized TRL standards were measured, and their S-parameters were stored for an offline post-processing stage. Then, the coplanar structures were measured twice, with the AMC structure (GapCPW) and with the bulk metallic ground (CB-CPW). An offline TRL algorithm was implemented in MATLAB to de-embed the effect of the GSG probes in the measurements and obtain the actual S-parameters of each line.

**Figure 17:**
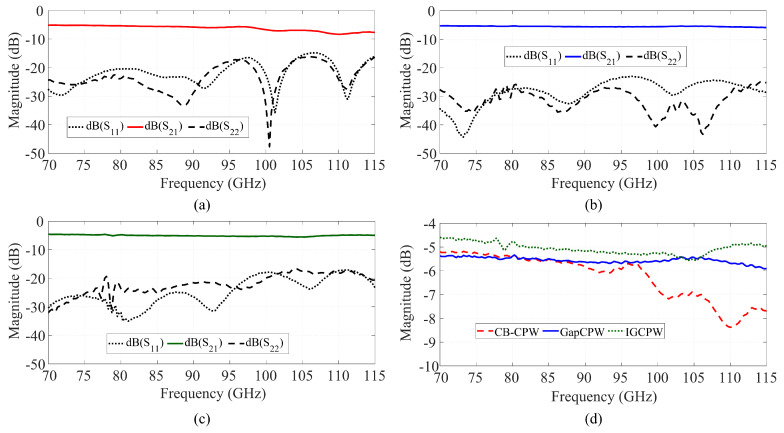
Comparison of the S-parameters of a sample 5-mm-long coplanar line. (**a**) CB-CPW. (**b**) GapCPW. (**c**) IGCPW. (**d**) Comparison of the S_21_.

Figure 17 shows the measured S-parameters of a sample, 5-mm-long line with 250 μm lateral ground extension after TRL de-embedding. Figure 17a shows the measurements for the CB-CPW case (bulk aluminum block below the substrate). In Figure 17b, the same line was measured again, replacing the aluminum block by the fabricated AMC structure (open GapCPW). Lastly, the top encapsulation was placed and aligned manually with the built-in microscope in the probe station to implement the IGCPW. This measurement is shown in Figure 17c. In addition, Figure 17d provides a comparison of the $S_{21}$ in the three cases.

Overall, the influence of substrate modes in the CB-CPW is clearly observable, especially in the standing wave being formed in the $S_{21}$ and the lower transmission at higher frequencies. According to Figure 6, it can be checked that the next higher order mode starts propagating at above 123 GHz. Therefore, this poorer performance in the line is due to the TM_0_, which has no cut-off frequency. On the other hand, the S_21_ in the GapCPW does not seem affected by this mode, showing an overall higher transmission, from 80 GHz to the end of the band. The poorer performance at the start of the band can be traced back to Table 1, where it was shown that the operational band is reduced from its lower end when the lateral ground is less than 2 pin periods. The small ripples between 75 and 80 GHz give evidence of the beginning of the periodic bed of pins to work as an AMC. Finally, the IGCPW shows a generalized enhanced transmission, as expected from the previous study, due to a strong interaction of the encapsulation with the central conductor, that allows propagating a significant amount of the power over the air.

## 7. Discussion

Due to the thin thickness of the wafer, its mechanical stability was compromised during the measurement campaign, and the lines with wider coplanar ground planes could not be measured. Such lines, according to the study presented in Section 4 and Table 1, should present an increased bandwidth of operation, extending the lower end of the band. Nevertheless, the measurements shown in the previous section provide enough evidence to validate the previous eigenmode study, showing an enhanced transmission thanks to the prevention of substrate modes. In addition, the excellent S_11_ measured for the open GapCPW validates the expressions provided in Section 3 for the characteristic impedance, which were used in this experiment to design the dimensions of the coplanar lines.

**Figure 18:**
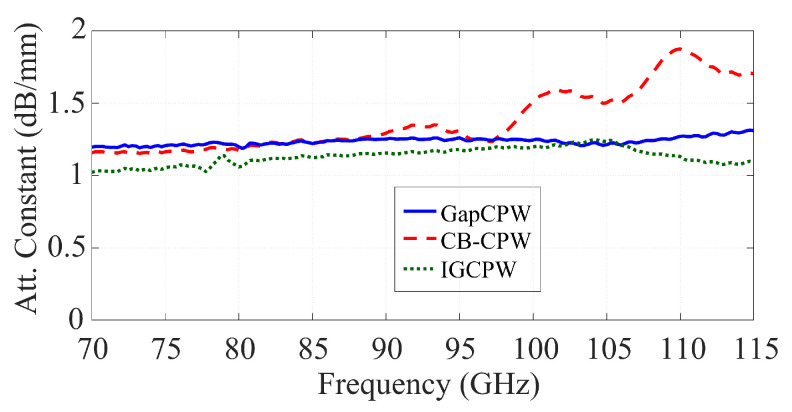
Extracted attenuation constant.

**Figure 19:**
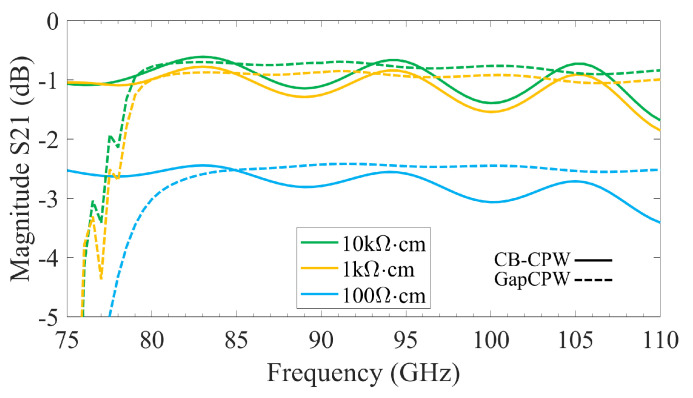
W–band Simulated S_21_ Parameters in HFSS for 5-mm-long lines with varying resistivity.

As for the measured transmission, it must be noted that the main goal of the experiment was to validate the concept and to prove that the GapCPW offers a substrate-mode-free, lower-loss transmission than the CB-CPW. This was shown in Figure 17d. Here, the S_21_ of the CB-CPW is affected by standing waves due to the TM_0_ mode, whereas the GapCPW is not. The open GapCPW does not show an enhancement in the transmission at the lower end of the band, because the stopband is not yet defined. At higher frequencies, the reduction in the propagation loss is masked by the standing waves of the TM_0_ mode. In addition, the IGCPW shows an enhanced transmission of more than 15%, especially at the lower end of the band. This is because the metallic cover extends farther than the lateral grounds of the GapCPW, contributing to defining the stopband together with the AMC earlier than the GapCPW. This can also be observed in Figure 18, where the attenuation constant α is extracted from the TRL de-embedded results (the Thru standard was 300 μm long). The extraction of this parameter is straightforward, as the magnitude of the $S_{21}$ parameter can be expressed by an exponential term, namely $|S_{21}|=e^{-\alpha \cdot L}$, where *L* is the length of the measured lines minus the length of the Thru employed in the calibration. The motivation of this work to provide a proof-of-concept must be borne in mind, since the measured α is rather high for a practical application. Nevertheless, it is straightforward that the use of a substrate with a higher resistivity (lower tanδ) will produce a lower attenuation while keeping the benefits of employing the AMC structure to prevent substrate modes and reduce the dielectric loss relative to the CB-CPW. This has been simulated in HFSS and is shown in Figure 19, where the S_21_ is provided for different resistivity values. Here, it can be seen that the undulation in the transmission parameter is prevented by the GapCPW configuration.

As for the measured attenuation, of about 1.2–1.3 dB/mm, this value can be traced back to the study performed in Section 5 concerning the conductor and dielectric loss in GapCPW. As a quick calculation, assuming a conductor loss twice as the one provided in Figure 10 (where the DC conductivity was used), an approximated dielectric loss of about 1–1.1 dB/mm is obtained, which is about one-third of the attenuation shown in Figure 12a for a resistivity of 10 Ω·cm. Correlating this with Equation (12), it can be concluded that the resistivity of the wafer employed in the experiment lies within the upper end of the range specified by the supplier (30 Ω·cm).

All in all, this work opens up a plethora of further research lines, such as the integration of active components (sources, diodes, oscillators), which are traditionally implemented in coplanar waveguide technology, where the use of the AMC removes the necessity of including vias along the perimeter of the line to prevent the substrate modes. Another line for future research lies in the development of transitions to other waveguide technologies, with a special focus on the transition to other technologies in the Gap Waveguide family, where the implementation of the AMCs for the coplanar and the groove/ridge gap waveguide parts might be unified in a single manufacturing process. Special attention shall be paid to the development of encapsulation solutions for the IGCPW configuration, so that the alignment of the channels with the CPW traces is facilitated. In addition, since the GapCPW has the potential to reduce the loss caused by the dielectric substrates, depending on the power budget of a system, cheaper PCB materials (with typically poorer electrical performances) may be selected to implement their circuit traces with GapCPW technology. Finally, this technology could be beneficial to any application in which substrate modes limit the performance, such as in sensing [28,30,31,32,33] or material characterization applications [34,35] relying on coplanar waveguide technology and whose bandwidth of operation can be limited by the excitation of these undesired modes.

Lastly, it is worth discussing the applicability of this technology at each frequency regime. In the lower part of the microwave range (L, S, and C bands), the processes for realizing the metallic vias are mature enough to offer a cost-effective solution. On the other hand, the realization of the AMC at these frequencies could be somewhat bulky, and the dielectric loss of the materials is often not critical. As such, it is unlikely that this technology may succeed in this regime. As for the THz frequencies (above 300 GHz), existing technologies with extremely thin dielectric layers may impose challenging conditions to support the dielectric substrate on top of the AMC. In addition, the increasing losses at THz frequencies due to conductor materials become more critical than the dielectric losses, making the adoption of this technology in such a frequency regime also unlikely. Where the GapCPW technology could really offer greater benefits is in the intermediate region, the upper bands (X, Ku, Ka) of the microwave frequencies and the mmWave range (30–300 GHz). At these frequencies, substrate modes increasingly impose serious limitations on the systems, and the realization of vias starts being more challenging due to their miniaturization. In addition, conductor losses in this regime are not as critical as in the THz bands, and, therefore, minimizing the dielectric losses with the GapCPW technology could be a key factor in the development of more efficient mmWave systems.

## 8. Conclusions

In this work, the concept of Gap Coplanar Waveguide and its different configurations has been studied in depth and validated experimentally. Closed-form expressions to compute the characteristic impedance and effective permittivity of the lines have been provided. In addition, a comprehensive study on the different modes propagating in the line and the loss associated with its fundamental mode has been performed. All this has been supported with an actual design, fabrication, and measurement of a prototype, supporting and validating the theoretical contributions provided in simulation. Finally, the applicability of this technology has been discussed and guidelines for future steps were provided.

## Figures and Tables

**Table 1:** Effect of the lateral ground extension.

k = 4.44 rad/mm	CB-CPW		GapCPW				
GND ext. (μm)	$\epsilon_{r}$ Even	$\epsilon_{r}$ Odd	$\epsilon_{r}$ Even	$\epsilon_{r}$ Odd	$f_{\mathrm{LOM}}$	$f_{\mathrm{HOM}}$	BW (%)
50	6.43	7.02	6.23	6.04	78.7	114.1	36.72
150	6.59	7.88	6.10	5.56	78.7	119.8	41.40
250	6.68	8.57	5.99	5.52	78.0	123.2	44.93
500	6.97	9.7	5.95	5.06	77.4	126.1	47.86
1000	N/A	N/A	5.95	5.03	62.7	126.6	67.51
1500	N/A	N/A	5.94	5.02	60.2	126.6	71.09

**Table 2:** Effect of the lateral ground extension in the operational bandwidth and odd mode cut-off.

k = 4.44 rad/mm		$w_{c}$						
$h_{c}$ (μm)	Freq. (GHz)	300	350	400	450	500	550	600
50	$f_{\mathrm{LOM}}$	60.4	60.1	60.0	60.1	60.5	60.2	60.2
	$f_{\mathrm{HOM}}$	162	164	161	160	159	157	156
	${\%}_{\mathrm{BW}}$	91.4	91.4	91.4	90.8	89.7	89.1	88.6
	$f_{\mathrm{odd}}$	134	130	123	118	115	111	107
	$\epsilon_{\mathrm{eff}}$	5.13	5.15	5.26	5.28	5.29	5.34	5.36
100	$f_{\mathrm{LOM}}$	60.4	60.2	59.8	60.0	60.4	59.7	60.0
	$f_{\mathrm{HOM}}$	151	148	145	143	141	140	139
	${\%}_{\mathrm{BW}}$	85.7	84.3	83.2	81.8	80.0	80.4	79.4
	$f_{\mathrm{odd}}$	107	101	97	93	90	86	84
	$\epsilon_{\mathrm{eff}}$	5.76	5.82	5.84	5.87	5.89	5.91	5.91
150	$f_{\mathrm{LOM}}$	58.9	59.9	59.8	60.0	59.9	59.7	59.9
	$f_{\mathrm{HOM}}$	142	139	137	136	134	133	133
	${\%}_{\mathrm{BW}}$	82.7	79.5	78.5	77.4	76.4	76.1	75.8
	$f_{\mathrm{odd}}$	92	87	83	80	77	74	72
	$\epsilon_{\mathrm{eff}}$	5.90	6.00	6.01	6.02	6.04	6.05	6.05
200	$f_{\mathrm{LOM}}$	60.0	60.0	60.1	60.1	60.2	60.2	60.2
	$f_{\mathrm{HOM}}$	138	135	134	132	131	131	130
	${\%}_{\mathrm{BW}}$	78.8	76.9	76.1	74.9	74.3	74.1	73.7
	$f_{\mathrm{odd}}$	83	78	75	72	69	68	65
	$\epsilon_{\mathrm{eff}}$	5.97	6.04	6.04	6.06	6.08	6.08	6.09
250	$f_{\mathrm{LOM}}$	59.9	60.0	60.0	60.1	59.8	60.2	59.9
	$f_{\mathrm{HOM}}$	135	133	131	130	129	129	129
	${\%}_{\mathrm{BW}}$	77.1	75.6	74.3	73.5	73.3	72.7	73.2
	$f_{\mathrm{odd}}$	77	72	69	67	65	63	62
	$\epsilon_{\mathrm{eff}}$	5.98	6.01	6.07	6.08	6.09	6.09	6.10
300	$f_{\mathrm{LOM}}$	59.7	59.9	59.8	60.1	59.5	60.0	60.3
	$f_{\mathrm{HOM}}$	133	132	130	129	129	128	128
	${\%}_{\mathrm{BW}}$	76.1	75.1	74.0	72.8	73.7	72.3	71.9
	$f_{\mathrm{odd}}$	71	67	65	63	61	60	59
	$\epsilon_{\mathrm{eff}}$	6.00	6.03	6.07	6.09	6.10	6.11	6.12

## Data Availability

Not applicable.
